# Protection motivation theory in predicting intentional behaviors regards schistosomiasis: a WeChat-based qualitative study

**DOI:** 10.3389/fpubh.2024.1295081

**Published:** 2024-05-28

**Authors:** Yi Wang, Chengyuan Li, Jianfeng Zhang, Yuanchun Mao, Wei Li

**Affiliations:** National Health Commission Key Laboratory of Parasitic Disease Control and Prevention, Jiangsu Provincial Key Laboratory on Parasite and Vector Control Technology, Jiangsu Provincial Medical Key LaboratoryJiangsu Institute of Parasitic Diseases, Wuxi, China

**Keywords:** behavior modification, schistosomiasis, education, protection motivation theory, behavior change

## Abstract

**Background:**

Modifications of behavior can help reduce the risk of transmission by disrupting the parasite life cycle. Behavior intension is a necessary intermediate step in behavior change. This study aimed to explore protection motivation theory (PMT) in predicting likelihood of engagement in protective behavior against infection with Schistosoma.

**Methods:**

In China, a questionnaire for data collection was sent to users who followed the WeChat public account from June 2 to 6, 2023. Factors affecting intentional behavior of participants were analyzed using stepwise regression analysis and structural equation modeling.

**Results:**

A total of 2,243 valid questionnaires were collected, with a mean age of 30 ± 8.4 years. Approximately 1,395 (62.2%) participants reported that they had been exposed to wild waters in daily work and life. About 51.0 and 50.7% of respondents reported never having been exposed to wild water in the last 3 and 6 months, respectively. Results indicated that prior knowledge of schistosomiasis was associated with the 7 PMT subconstructs, which then influenced future preventative behaviors.

**Conclusion:**

Behavior intentionis a complicated and indispensable part of behavior change that is influenced by professional knowledge, socio-economic status, and personal characteristics. The effective dissemination of knowledge regards schistosomiasis should be strengthened to emphasize the effectiveness of protective measures against infection and severe disease.

## Introduction

Schistosomiasis, caused by *Schistosoma japonicum (S. japonicum)*, was highly prevalent in China before the initiation of the national schistosomiasis control program in the mid-1950s (1). The symptoms of schistosomiasis include abdominal pain, diarrhea, bloody stools, fever, enlarged spleen or liver, liver fibrosis, portal hypertension, and fluid accumulation in the peritoneal cavity (2). Remarkable progress in schistosomiasis control has been achieved in China since the launch of the national program. Only 30,000 prevalent schistosomiasis patients were estimated across the country, with only five new infections detected in 2019 (3). However, a total of 53,254 existing habitats for the vector were identified, with three clusters in the Sichuan Basin, Dongting Lake, and Poyang Lake. These snail habitats are spread across 12 provinces covering an area of 3.58 billion m^2^, where *Oncomelania hupensis* (*O. hupensis*) was identified through a nationwide survey in 2016 (4). Schistosomiasis can be acquired after exposure to freshwater cercariae released from *O. hupensis* (5). Consequently, these widespread intermediate hosts remain an important source of infection, particularly as the World Health Organization (WHO) aims to eliminate the disease in all endemic countries by 2030 (6). Thus, schistosomiasis remains a serious public health issue in China.

Various control measures have been developed and utilized over.

the past few decades, including snail control, personal hygiene attention, drug administration, improved sanitation, access to safe water, and health education (7). In February 2022, the WHO launched a new guideline for action against human schistosomiasis. This guideline suggests that strategies should include preventive chemotherapy, focal snail control, case management, health education, and behavior change (BC) interventions (8). Changing people’s behavior can disrupt the life cycle of parasites and reduce the risk of transmission. Health education is a common strategy for BC, particularly focusing on individual-level improvements in knowledge of schistosomiasis. A previous study involved an assessment of 32 BC schistosomiasis interventions for shcistosomiasis (9), reporting a significant reduction in risky practices and a large increase in preventive measures after implementing such interventions. Although BC was not the primary objective, it played an important and ongoing role in schistosomiasis control (10). Another study suggested that knowledge activates a belief system, affecting emotions, and subsequently leading to an intention to perform a specific behavior (11). Therefore, behavior intention is a necessary intermediate step in BC initiatives.

The protection motivation theory (PMT) is a classical framework aimed at explaining and predicting behavior intention through threat and coping appraisal. Threat appraisal involves an individual’s belief about the negative consequences of health threat (perceived severity), their vulnerability to these consequences (perceived vulnerability), and the benefits of the performance of the maladaptive behavior (intrinsic and extrinsic rewards). Coping appraisal is determined by beliefs about the effectiveness of preventative behavior (response efficacy), confidence in one’s ability to perform the behavior (self-efficacy), and barriers to performing the behavior (response costs). Motivation, an important determinant of protective behavior, is often correlated with intention (12). Therefore, cognitive predictors such as severity, vulnerability, response efficacy, and self-efficacy are associated with intentions, serving as mediators of BC. PMT has shown moderate success in predicting health-related intentions and behaviors across contexts, including COVID-19 vaccination (13), diabetes mellitus type 2 (14), cervical cancer (15), schistosomiasis (16), and others. According to the most recent WHO program guidelines, effective BC is crucial for achieving the 2030 NTD roadmap goals (17). Our study aimed to explore PMT’s role in predicting intention to engage in protective behaviors against schistosomiasis.

## Materials and methods

### Study design

**Figure 1:**
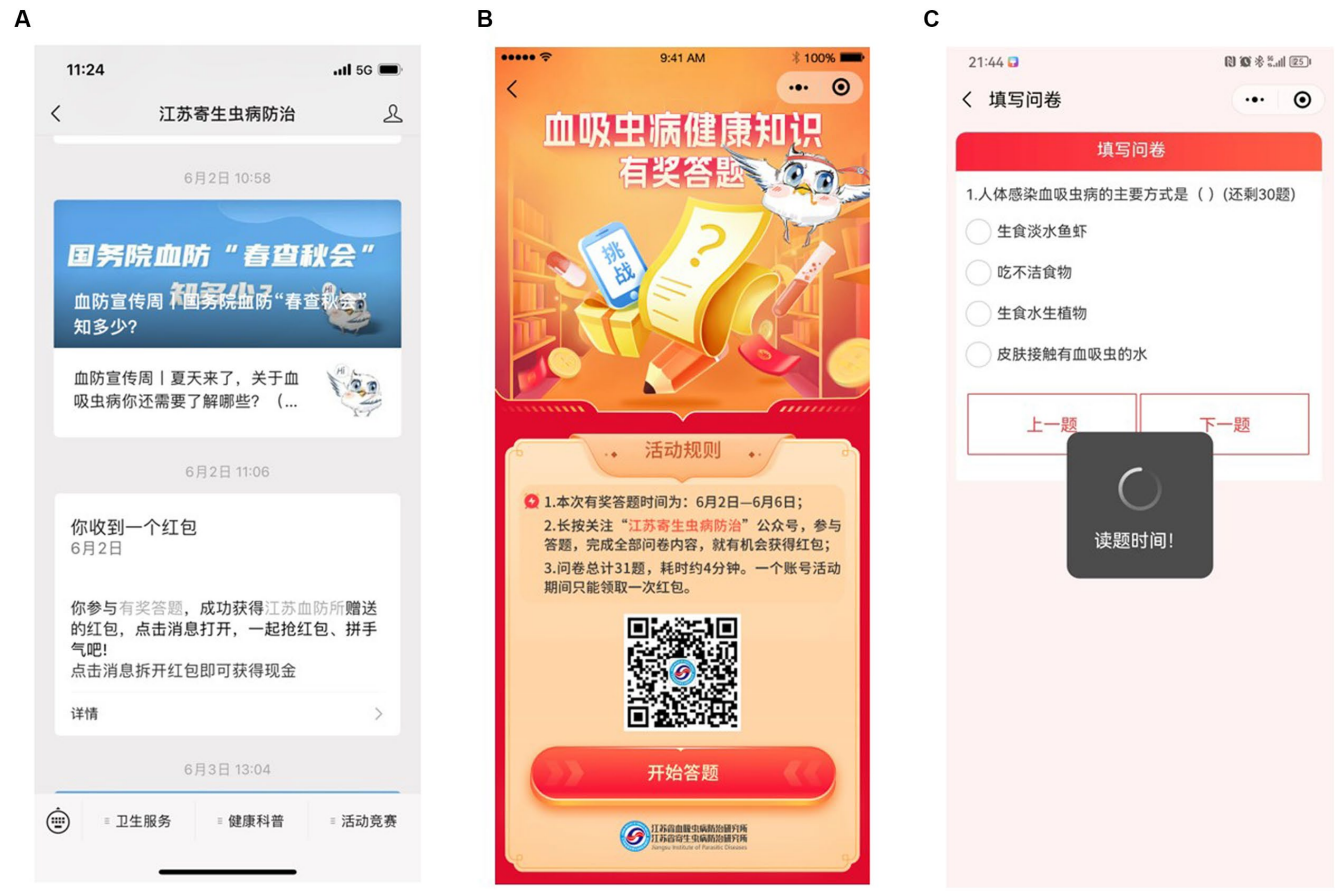
The activity interface. **(A)** Frontpage of the WeChat public account; **(B)** entry to the survey; **(C)** questionnaire interface.

This activity was conducted using a free online survey tool, the Jiangsu Institute of Parasite Disease’s official WeChat account. Health information on parasitic diseases was available to followers on the account by JIPD since 2016. A questionnaire for data collection was sent via the official WeChat account from June 2 to 6, 2023 (Figure 1).

### Questionnaire development

The questionnaire was entitled “Questionnaire on knowledge of schistosomiasis” and comprised 31 questions including demographics, schistosomiasis knowledge, awareness of schistosomiasis and exposure to wild water, previous protective behaviors and future behavior, and the schistosomiasis PMT scale (Supplementary file 1).The questionnaire was developed based on the constructs of the PMT model (Rogers, 1983) and related schistosomiasis topics such as schistosomiasis spread, schistosomiasis pathology, and prevention of schistosomiasis. Seven single-choice questions were used to assess participant knowledge of schistosomiasis. Participants earned one point for each correct response. Participants were asked for their awareness of schistosomiasis and exposure frequency to wild water through 5 questions, using 4 choices coded as follows:(0 = never, 1 = occasionally,2 = of tenor monthly, 3 = always or weekly). To measure past protective behaviors and future protective behaviors, four questions were asked using a 4-levelresponse scale: (0 = never, 1 = occasionally, 2 = often, 3 = always) and a 5-levelresponse scale (1 = very unlikely to 5 = very likely). The PMT scale consisted of 15 items assessing the7 PMT subconstructs using a 5-point Likert scale (1 = completely agree to 5 = completely disagree).

### Statistical analysis

Data including demographics and scores were collected when the questionnaires were submitted. The factors affecting participant behavior intention were analyzed using stepwise regression analysis. Structural equation modeling (SEM) was performed to analyze the relationships of the questionnaire variables. SEM was assessed using 4 indices: goodness-of-fit index (GFI) >0.9, comparative-fit-index (CFI) >0.9, root mean square error of approximation (RMSEA) <0.05, and χ^2^/df < 3 [17]. Ap value <0.05 was considered statistically significant. Statistical analysis was completed using IBM SPSS, version 25.0 (Chicago, United States). An online statistics tool named SPSSAU (https://spssau.com/index.html) was used for structural equation modeling (SEM) and mediation analysis.

## Results

### Participant characteristics

A total of 2,243 valid questionnaires were collected from the network.1302 (58%) were male and 941 (42%) were female, with a mean age of 30 ± 8.4 years. Participants in the survey had a high educational level, and more than half received an education beyond school level. Migrant workers, civil servants, and enterprise personnel comprised the main population at 80.8%. 35%participants said that they had suffered from schistosomiasis.Approximately1395 (62.2%) of participants reported that they had been exposed to recreational or wild waters through daily work and life (Table 1).

**Table 1:** Participants characteristics.

		Total *n* (%)			Total *n* (%)
Gender			Career		
	Male	1,302 (58.0)		Farmer	290 (12.9)
	Female	941 (42.0)		Migrant workers	487 (21.7)
Education				Civil servant	769 (34.3)
	Primary school	112 (5.0)		Enterprise personnel	557 (24.8)
	High school	510 (22.7)		Student	141 (6.3)
	Bachelor’s degree	1,321 (58.9)			
	Master’s degree or above	300 (13.4)			

### Descriptive statistics of the questionnaire

**Figure 2:**
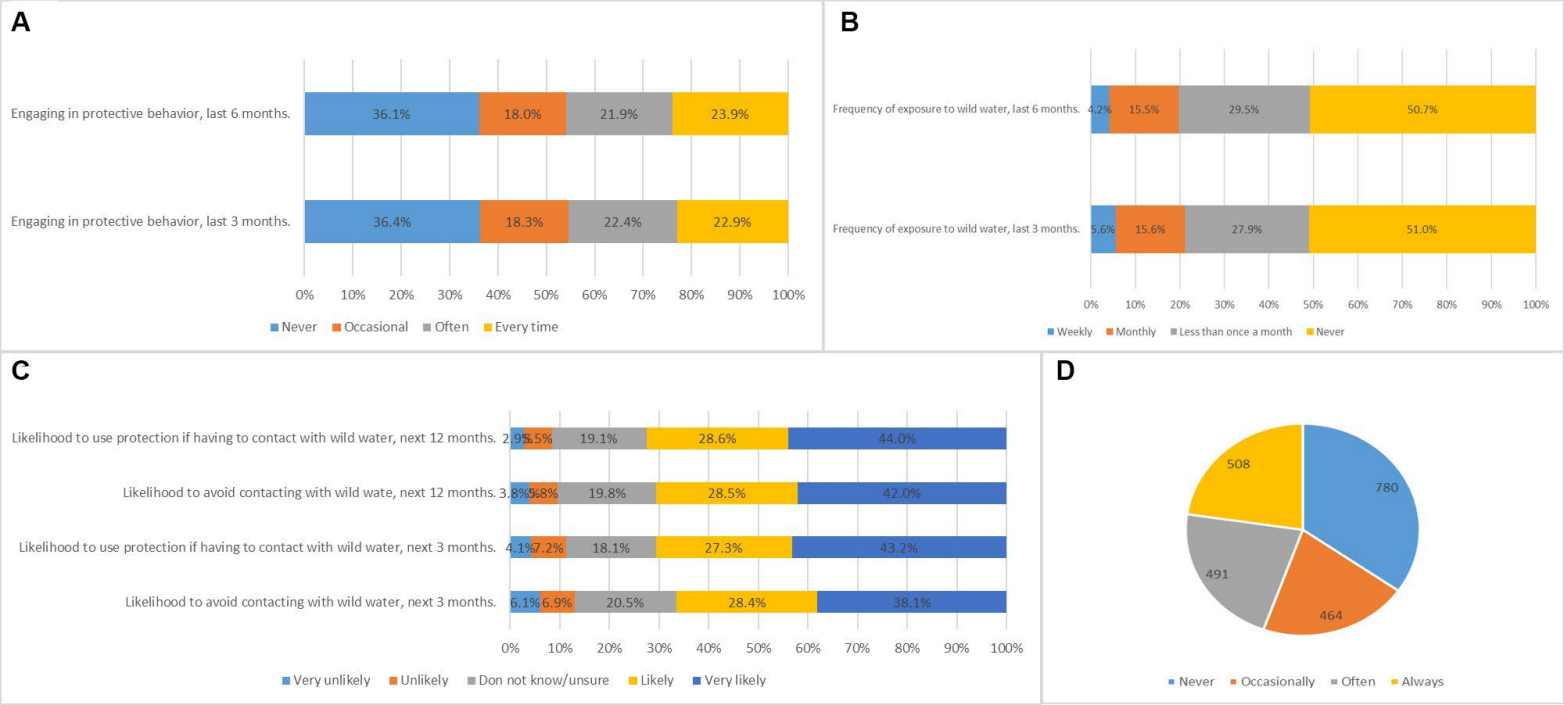
**(A)** Characteristics of engaging in protective behavior, last 3 and 6 months. **(B)** Frequency of exposure to wild water, last 3 and 6 months. **(C)** Characteristics of future protective behaviors. **(D)** Awareness of schistosomiasis before exposure to wild water.

The reliability of PMT scale was 0.738 (Cronbach’s *α*) and construct validity was 0.926 (KMO). The reliability of two subconstructs named threat and coping appraisal of PMT scale were 0.828, 0.739 (Cronbach’s *α*) and construct validity were 0.901, 0.718 (KMO). The mean score for schistosomiasis knowledge was 3.0 ± 1.7 (the total score possible was 8 points). Only 20.7% of participants reported that they were always aware of schistosomiasis before exposure to recreational water and 34.8% said they had never been aware of it before exposure.51.0 and 50.7%respondents reported that they had never been exposed to wild water in the last 3 and 6 months, respectively. Approximately 38.1 and 42.0%ofparticipants reported that they were likely to avoid contact with wild water in the next3 and 12 months, respectively. The descriptive statistics from the questionnaire are presented in Figure 2.

### Association between questionnaire variables and behavior intention

**Figure 3:**
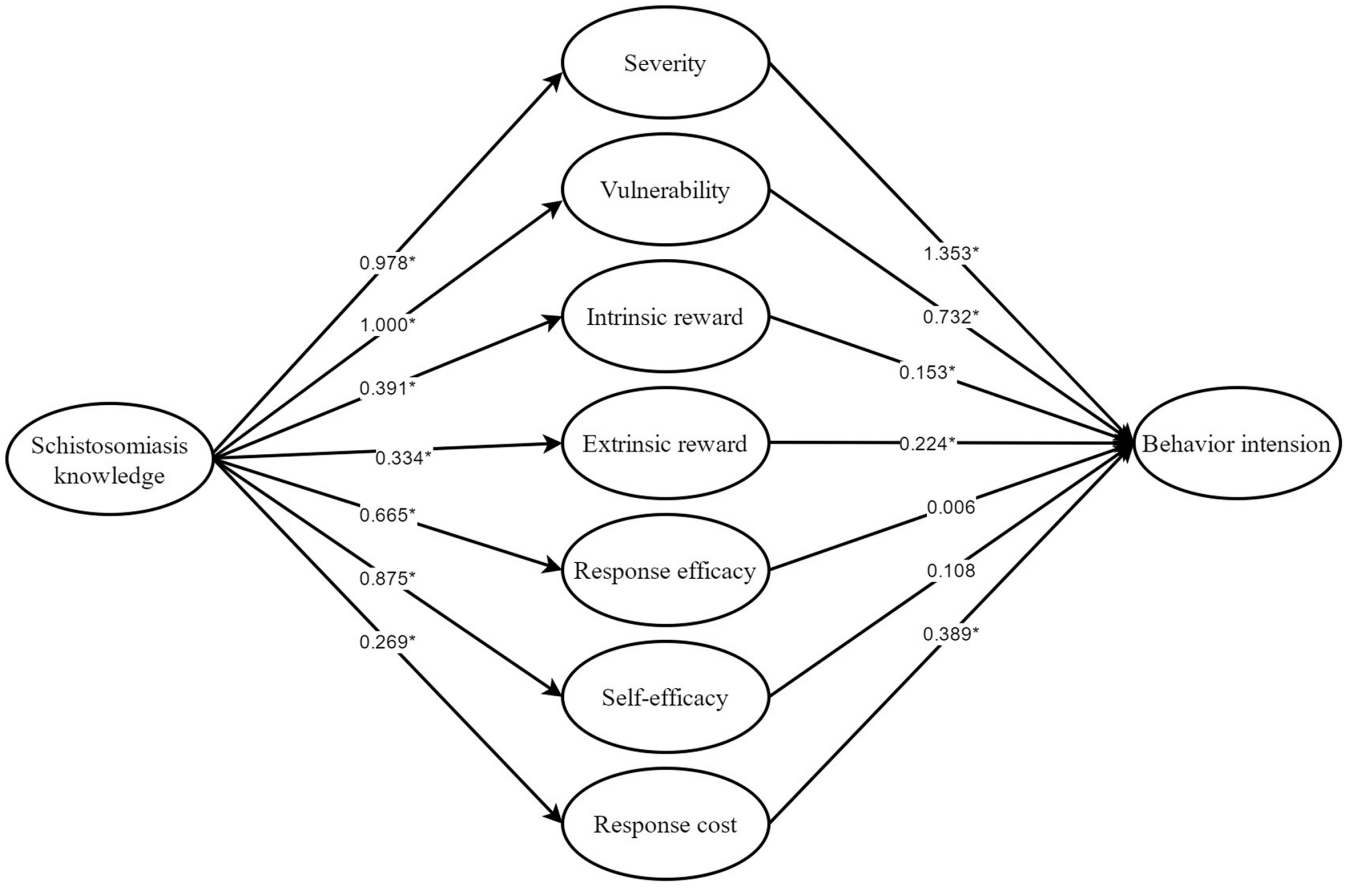
Structural equation modeling of schistosomiasis knowledge, schistosomiasis PMT constructs and behavior intention. *p < 0.05.

The stepwise regression analysis was used to analyze participant behavior intention. The main factors were gender, schistosomiasis knowledge, prior exposure, and prior protective behavior (Table 2). The hypothesized model analysis of this study showed a relatively good fit (*χ*^2^/df = 3.0, GFI = 0.86, RMSEA =0.07, CFI =0.87). Results indicated that schistosomiasis knowledge was associated with the 7 PMT subconstructs, which then significantly influenced future protective behaviors (behavior intention). All parameters of SEM are illustrated in Figure 3.

**Table 2:** Results of the stepwise regression analysis.

Factors	B	*S.E*	T	*P*
Constant	14.757	0.245	60.336	0.000
Prior exposure	0.427	0.037	11.711	0.000
Prior protective behavior	−0.568	0.043	−13.340	0.001
Gender	−2.994	1.316	−2.276	0.012
schistosomiasis knowledge	0.109	0.049	2.219	0.027

*B, unstandardized regression coefficient; SE, standard error; *p* < 0.05.

## Discussion

People can become infected with schistosomiasis by contact with freshwater harboring cercariae released from intermediate host snails. Therefore, schistosome infection is closely related to human behavior (18). Behavior change can improve schistosomiasis control by modifying risky practices (19). PMT is a theory that analyzes the cognitive process of behavior intention from the perspective of motivation. Behavior intentionis a decision made through a comprehensive assessment of both the threat of risk factors and the ability to coping with these and has been predicted widely using PMT. A pilot study of WeChat-based using PMT was conducted to predict behavior intentions against schistosomiasis.

Interventions combining information provision was an important part of BC. Despite adequate knowledge and positive attitudes, this did not translate to effective behavior changes, mainly due to the socio-cultural factors and participant environment. It is crucial to understand the social, cultural, and behavioral determinants that can bridge the knowledge-practice gap (20). According to our study, participant behavior intention was strongly associated with female gender, schistosomiasis knowledge, prior exposure, and prior protective behavior. Women were more likely than men to take protective measures. People who had been exposed to wild or recreational waters in the past were less likely to take protective measures in the future. One reason for this phenomenon may be survivorship bias (21). Even if these participants were exposed to wild waters, they were not infected with schistosomiasis. Therefore, they would not choose to take protective measures in the future. People who had taken protective measures in the past and achieved a high score on schistosomiasis knowledge were more likely to do so in the future. These finding showed that behavioral determinants should not only focus on knowledge transmission but also on early and acquired living habits.

As a result of the gaps observed between knowledge, attitude, and practices, more effective methods and considerable effort should be invested to better analyze the reasons for what motivates behavior change (22). The PMT framework for systematic behavior involved three phases: (1) assessing the risks; (2) benefiting from take protective measures; (3) making a decision. The result of the structural equation modeling analysis indicated that schistosomiasis knowledge was associated with all PMT subconstructs. Five of the seven subconstructs (severity, vulnerability, intrinsic reward, extrinsic reward, and response cost) were significantly associated with intention to engage in protective behavior. These results accentuated the necessity of schistosomiasis knowledge for subsequent behavior intention.

Focusing more on the emerging model, results indicated that schistosomiasis knowledge was significantly associated with four subconstructs including severity, vulnerability, self-efficacy, and response efficacy. Meanwhile, weaker associations were observed with intrinsic reward, extrinsic reward, and response cost. Severity and vulnerability were recognized as important clinical features of the disease. Response efficacy referred to the understanding of disease prevention and information concerning control. In most of these studies, the knowledge, attitudes, and practices (KAP) questionnaire on schistosomiasis control also included cause of infection, symptoms, prevention, and risky behaviors (23–25). Furthermore, not all the four substructures were closely associated with knowledge, which was associated with behavior intention. For example, self-efficacy represented confidence in one’s ability to take protective behaviors. Moreover, three dimensions including intrinsic reward, extrinsic reward, and response cost were a comprehensive embodiment of personal cognition, social status, and culture. The three dimensions were weakly correlated with knowledge and strongly associated with behavior intention. So, for behavior intention, severity and vulnerability were the most important factors, which in turn were associated with knowledge of disease. However, another study suggested that severity, intrinsic reward and self-efficacy should be targeted (26, 27). It is possible that because this study focused on participants whose mean age was 30 years old, while the previous study focused on students whose mean age was 13 years old.

By and large, in combining BC approaches for schistosomiasis intervention programs, the content of behavior intention should be carefully considered. First, the characteristics of the target population should include age, gender, and early living habits. These appear to be important considerations for health education campaigns as part of disease control interventions. Second, the effective dissemination of information regarding schistosomiasis should be strengthened to consolidate knowledge on disease severity, vulnerability, and emphasis on the effectiveness of protective measures. Third, there is a high correlation between response costs and behavior intention, and this accentuates the need to develop economic and convenient protection to foster behavior intention.

Our study has some limitations. First, all participants may have had a higher socioeconomic background than the general public and thus are not representative of the entire population. To be able to generalize the results, participants who do not have access to smartphones should be included in the future. Second, response bias might be present because participant self-reporting of responses to questions about their capacity for healthy behaviors and skills might have been biased toward their responses and what the study investigators thought of these. Third, one individual may possess multiple phone lines connected to the WeChat platform, enabling them to provide duplicate responses in order to intentionally skew the data when contacted without their knowledge.

## Conclusion

Behavior intentionis a complicated and indispensable component of behavior change that is influenced by professional knowledge, socio-economic status and personal characteristics. PMT highlighted that behavior intention and knowledge were inextricably linked in numerous ways and can mitigate against schistosomiasis infection.

## Data availability statement

The original contributions presented in the study are included in the article/Supplementary material, further inquiries can be directed to the corresponding authors.

## Ethics statement

The studies involving humans were approved by the Ethics Committee of the Parasitic Disease Control and Prevention in Jiang. The studies were conducted in accordance with the local legislation and institutional requirements. The participants provided their written informed consent to participate in this study.

## Author contributions

YW: Project administration, Investigation, Writing – original draft. CL: Writing – review & editing, Investigation, Data curation. JZ: Writing – review & editing, Project administration. YM: Formal analysis, Writing – review & editing, Supervision. WL: Writing – review & editing, Project administration.
