# Supplementary material for: Protection motivation theory in predicting intentional behaviors regards schistosomiasis: a WeChat-based qualitative study
Source: Front Public Health. 2024 May 28;12:1295081. doi: 10.3389/fpubh.2024.1295081 (PMC11165043; doi:10.3389/fpubh.2024.1295081)
Supplement: Supplementary file 1 [file Data_Sheet_1.docx]

**Schistosomiasis knowledge**

1. How is schistosomiasis spread?

①Eating raw zizania aquatica or eleocharis dulcis

②Eating dirty food and drinking dirty water

③Eating raw aquatic plants

④Exposure to freshwater harboring schistosomiasis

2. what is the meaning of “epidemic water”.

①Water were inhabited by oncomelania hupensis

②Water harboring cercariae

③All water in endemic areas

④Faecally contaminated water

3. Which of the following ways can be infected by schistosome?

①Touching a patient with schistosome

②Eating dirty food and drinking dirty water

③Mosquito bites

④Go to a place with oncomelania hupensis to wash clothes or swim

4. Which of the following symptoms are most likely to occur with schistosomiasis infection?

①High blood pressure

②Fever, abdominal pain, diarrhea

③Itchy all over the body

④Joint soreness

5.what is the main damage to the human body from schistosomiasis?

①Anaemia

②Hepatosplenomegaly

③Gastric ulcer

④Nervous breakdown

6. what is the best way to prevent schistosomiasis?

①Handwashing before eating and after excrement

②Don't eat dirty

③Don't go to a place with oncomelania hupensis to wash clothes or swim

④Vaccination

7. What measures should be taken to prevent and control schistosomiasis>

①Investigate and treat sick people and animals

②Treating feces, using water safely and protect yourself

③Eliminate oncomelania hupensis

④All of the above

**Awareness of schistosomiasis and exposure to water**

1.Awareness of schistosomiasis before exposure to wild water.

①Never

②Occasionally

③Often

④Always

2.Frequency of exposure to wild water, last 3 months.

①Weekly

②Monthly

③Less than once a month

④Never

3.Frequency of exposure to wild water, last 6 months.

①Weekly

②Monthly

③Less than once a month

④Never

4.Engaging in protective behavior, last 3 months.

①Never

②Occasional

③Often

④Every time

5.Engaging in protective behavior, last 6 months.

①Never

②Occasional

③Often

④Every time

**Previous protective behaviors and future behavioral intentions**

1.Likelihood to avoid contacting with wild water, next 3 months.

①Very unlikely

②Unlikely

③Don not know/unsure

④Likely

⑤Very likely

2.Likelihood to use protection if having to contact with wild water, next 3 months.

①Very unlikely

②Unlikely

③Don not know/unsure

④Likely

⑤Very likely

3.Likelihood to avoid contacting with wild wate, next 12 months.

①Very unlikely

②Unlikely

③Don not know/unsure

④Likely

⑤Very likely

4.Likelihood to use protection if having to contact with wild water, next 12 months.

①Very unlikely

②Unlikely

③Don not know/unsure

④Likely

⑤Very likely

**The Schistosomiasis PMT Scale**

**Severity**

1. Schistosomiasis causes big damage to people’s health.

①Definitely disagree

②Disagree

③Neutral

④Agree

⑤Definitely agree

2. It can lead to death infected with schistosomiasis if untreated promptly.

①Definitely disagree

②Disagree

③Neutral

④Agree

⑤Definitely agree

3.Quality of life will suffer if I get infected schistosomiasis.

①Definitely disagree

②Disagree

③Neutral

④Agree

⑤Definitely agree

**Vulnerability**

4. I will get infected if I play with or swim in wild water.

①Definitely disagree

②Disagree

③Neutral

④Agree

⑤Definitely agree

5. You will not be infected again, after you recover from schistosomiasis.

①Definitely disagree

②Disagree

③Neutral

④Agree

⑤Definitely agree

**Intrinsic Reward**

6.It is very enjoyable for me to play in water outside.

①Definitely disagree

②Disagree

③Neutral

④Agree

⑤Definitely agree

7. It is convenient to wash my hands and feet in rivers and ponds.

①Definitely disagree

②Disagree

③Neutral

④Agree

⑤Definitely agree

**Extrinsic Reward**

8. Playing in outdoor water with classmates is good for making friends and for strengthening friendship.

①Definitely disagree

②Disagree

③Neutral

④Agree

⑤Definitely agree

9. It will be more convenient to work or play in wild water without protective measures.

①Definitely disagree

②Disagree

③Neutral

④Agree

⑤Definitely agree

**Response Efficacy**

10. I will never be infected if I do not play in wild water.

①Definitely disagree

②Disagree

③Neutral

④Agree

⑤Definitely agree

11. I will never get infected if I use good protection measures before contacting with wild water.

①Definitely disagree

②Disagree

③Neutral

④Agree

⑤Definitely agree

**Self-Efficacy**

12. I can control myself not to go and play in wild water.

①Definitely disagree

②Disagree

③Neutral

④Agree

⑤Definitely agree

13. I can definitely say ‘‘no’’ even if my friends invite me to play in wild water.

①Definitely disagree

②Disagree

③Neutral

④Agree

⑤Definitely agree

**Response Cost**

14. My friends may tease me if I refuse to go out with them to play in wild water.

①Definitely disagree

②Disagree

③Neutral

④Agree

⑤Definitely agree

15. It will be not convenient to work or play in wild water with protective measures.

①Definitely disagree

②Disagree

③Neutral

④Agree

⑤Definitely agree
